# Malignant paraganglioma in children treated with embolization prior to surgical excision

**DOI:** 10.1186/s12957-016-0778-8

**Published:** 2016-02-02

**Authors:** Eduardo de Paula Miranda, Roberto Iglesias Lopes, Guilherme Philomeno Padovani, Paulo Renato Marcelo Moscardi, Fernanda Gardini Maciel Nishimura, Berenice Bilharinho de Mendonça, Francisco Cesar Carnevale, Lilian Maria Cristofani, Ricardo Jordão Duarte, Miguel Srougi, Francisco Tibor Denes

**Affiliations:** 1 Division of Urology, University of Sao Paulo School of Medicine, Sao Paulo, Brazil; 2 Department of Anesthesiology, University of Sao Paulo School of Medicine, Sao Paulo, Brazil; 3 Division of Endocrinology and Metabolism, University of Sao Paulo School of Medicine, Sao Paulo, Brazil; 4 Department of Radiology, University of Sao Paulo School of Medicine, Sao Paulo, Brazil; 5 Department of Pediatrics, University of Sao Paulo School of Medicine, Sao Paulo, Brazil

**Keywords:** Embolization, Paraganglioma/blood/genetics/surgery, Paraganglioma/diagnosis/mortality/surgery, Retroperitoneal neoplasms, Treatment outcome

## Abstract

**Background:**

Paragangliomas (PGL) are rare tumors derived from neural crest cells, whose origins may vary along the chain of the sympathetic nervous system. Such tumors are often characterized by secretion of catecholamines, but sometimes they are biochemically inactive, which makes diagnosis often challenging. Malignant paraganglioma is defined by the presence of this tumor at sites where chromaffin cells are usually not found or by local invasion of the primary tumor. Recurrence, either regional or metastatic, usually occurs within 5 years of the initial complete resection but long-term recurrence is also described. Malignancy is often linked to a SDHB mutation. Preoperative embolization has been applied in the surgical management of PGLs with the objective to decrease intra-operative blood loss and surgery length without complications.

**Case Presentation:**

We report two cases of patients with abdominal or pelvic malignant PGLs who have been treated surgically at our center after preoperative embolization. Surgery was a very challenging procedure with multiple surgical teams involved and embolization did not prevent major blood loss and intraoperative complications. Patients required adjuvant treatment with either chemotherapy or radiotherapy.

**Conclusions:**

Many studies in the adult population have established recommendations for the diagnosis and therapeutic management of PGL, but few studies concern the pediatric population. Because malignant PGL is more important in the pediatric population, screening and early diagnosis of PGL is advisable in children with genetic predisposing. Surgical resection is the mainstay of treatment, but a multimodal approach is often required due to the complexity of cases.  The role of preoperative embolization is not established and in our experience it has provided little benefit and major complications.

## Background

Paragangliomas (PGLs) are rare tumors derived from neural crest cells, whose origins may vary along the chain of the sympathetic nervous system. They may extend from the skull base to the mediastinum following the vagus nerve, but may also occur in the abdomen. Most of intra-abdominal PGLs present at the organ of Zuckerkandl, located on the left of the aorta near the origin of the inferior mesenteric artery or between the aorta and left renal vein. When these tumors manifest in the adrenal glands, they are classified as pheochromocytomas [1].

Such tumors are often characterized by secretion of catecholamines, but sometimes they are biochemically inactive, as in most head and neck PGLs. Often, the diagnosis is made incidentally after imaging or family screening performed in certain syndromes [2].

The incidence of PGL varies from 0.2 to 0.8 per 100,000 persons per year with a peak incidence between 30 and 40 years of age in the general population. The average age of diagnosis in the pediatric population is 11 years, but there are case reports as young as 5 years of age [3, 4]. There is a male predominance of 2:1 including adolescents. The frequency of bilateral tumors in children is twice as high as in adults (20 versus 5–10 %). About 10 to 20 % of all cases are diagnosed during childhood, and the incidence in the pediatric population is estimated at 0.011 per 100,000 children under 18 years of age, from which around 12 % are malignant [4, 5].

Malignant paraganglioma is defined by the presence of tumor in sites where chromaffin cells are usually not found or by local invasion of the primary tumor. The rate of metastatic disease is very variable in the literature, with reports ranging from 2.5 to 40 %. Recurrence, either regional or metastatic, usually occurs within 5 years of initial complete resection, but there are reports of recurrence up to 40 years postoperatively [3]. Although well described for the adult population, diagnosis and treatment of malignant pediatric paragangliomas are poorly described in the literature [4]. We report two cases of abdomino-pelvic malignant PGLs treated surgically at our center after preoperative embolization.

## Case presentation

### Case 1

An 11-year-old boy presented with a growing but painless pelvic mass for the past 2 years. He denied headaches or tachycardia and presented in good clinical conditions. He was submitted elsewhere to two surgical attempts of tumoral resection 12 and 6 months previously, both being unsuccessful due to diffuse bleeding. In the second procedure, a biopsy was performed, which revealed large cells with eosinophilic cytoplasm positive for S-100 and chromogranin, suggestive of PGL.

**Fig. 1:**
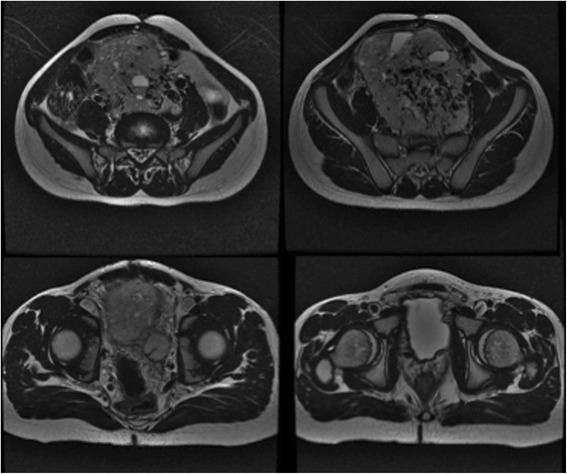
Abdominal MRI revealing a large mass with intense vascularization with close contact aorta and iliac bifurcation

**Fig. 2:**
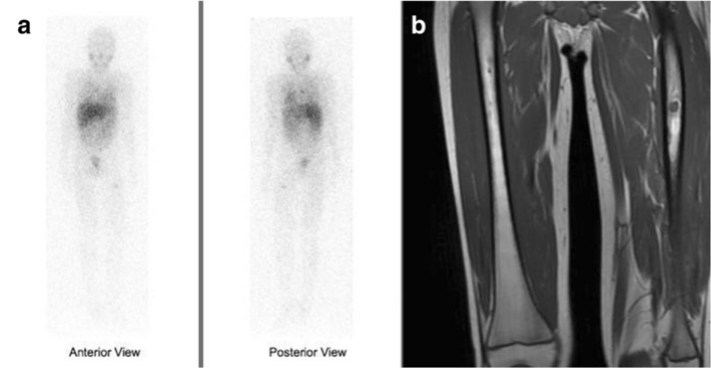
Scanning with 123-metaiodobenzylguanidine (I-123-MIBG) showing uptake on the proximal left femoral bone, suggestive of metastatic disease (**a**). Lower limb MRI revealing a proximal femoral lesion (**b**)

Physical examination was normal except for an infra-umbilical midline scar and a large abdominal mass visible and palpable in the hypogastric region. Laboratory exams were unremarkable, except for increased urinary excretion of noradrenaline (571 μg/24 h) and 4-OHmetoximandelate (13.2 mg/24 h). Abdominal ultrasound disclosed a well-defined retroperitoneal mass in the left paraaortic region below the renal vessels. Abdominal MRI revealed a large mass with intense vascularization, anterior to the aorta and iliac bifurcation (Fig. 1). Scanning with 123-metaiodobenzylguanidine (I-123-MIBG) revealed uptake on the proximal left femoral bone, suggestive of metastatic disease (Fig. 2a). A lower limb MRI was performed to examine the area of uptake (Fig. 2b).

**Fig. 3:**
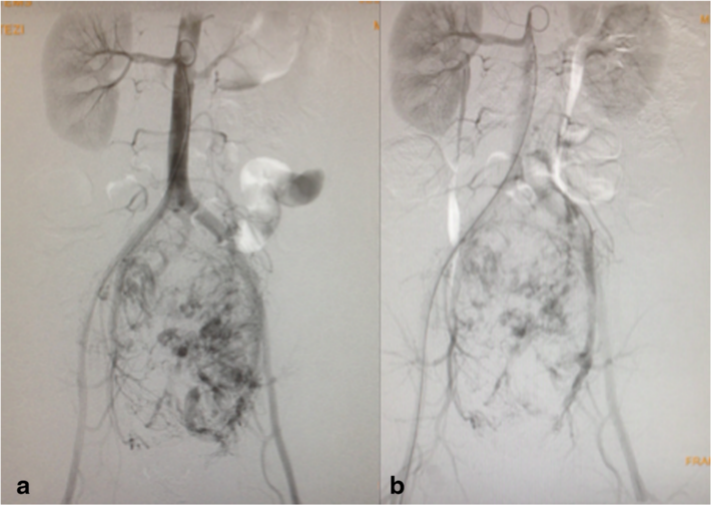
Arteriography showing arterial vascularization of the mass before (**a**) and after (**b**) the embolization

**Fig. 4:**
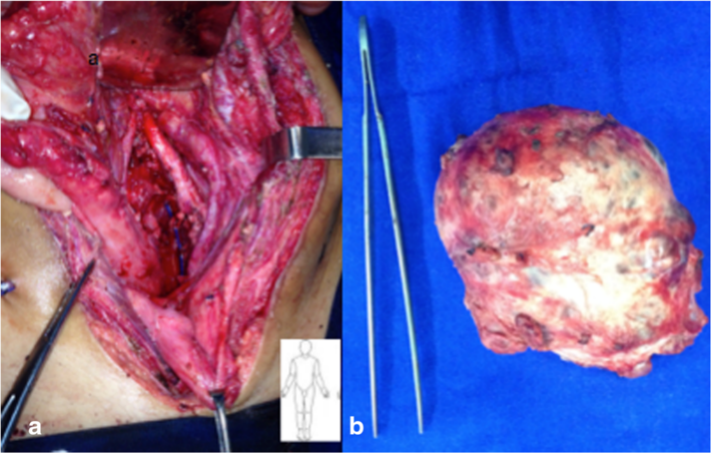
Pelvic aspect after mass removal with the iliac vessels in detail (**a).** Surgical specimen (**b**)

Preoperative embolization of two feeding vessels arising from the most caudal lumbar artery and branches of the left inferior iliac artery was performed. Radiological access was achieved by the catheterization of the right common femoral artery, and embolization was made with polyvinyl alcohol (PVA) and 700–1000 μ particles of *n*-butyl-2-cyanoacrylate 1:3. That resulted in a decrease of 50 % of the tumor vascularization (Fig. 3). After 4 days, he was submitted to a midline xiphopubic incision that provided good exposure of the tumor. Ureteral catheterization with double-J stent placed in the both ureters was performed just before skin incision. Dissection was challenging with massive bleeding despite the previous embolization. Intermittent left common iliac artery clamping was required, as well as definite ligation of the internal iliac artery, but the tumor was completely resected without intestinal or ureteral injury. Surgical specimen and site aspect after mass removal are shown in Fig. 4. The procedure lasted 9 h, and the patient received 16 packs of red blood cells. The postoperative procedure was uneventful with a minor stay at the intensive care unit, and the patient was discharged home on the 12th postoperative day, after removal of the ureteral stent.

Pathological evaluation revealed that the specimen measured 12.0 × 9.0 × 7.0 cm and weighed 463 g. Sectioning revealed a multilobular, heterogeneous tumor mass with foci of hemorrhage and necrosis. Microscopically, the tumor displayed intense cell pleomorphism and mild nuclear enlargement. Chromogranin A, synaptophysin, and S-100 stains were positive. Mitotic index was 3/10, there were no signs of extracapsular invasion but vascular invasion was confirmed.

After 6 months, the patient is in good health, without abdominal or urinary symptoms, but presented with paresthesia of the left thigh in the early postoperative period with no clinical improvement after 3 months. There is no sign of local abdominal recurrence. The patient received radiotherapy for the left femoral metastasis and is currently being treated with systemic chemotherapy. The patient was referred for genetic testing and awaits final results.

### Case 2

**Fig. 5:**
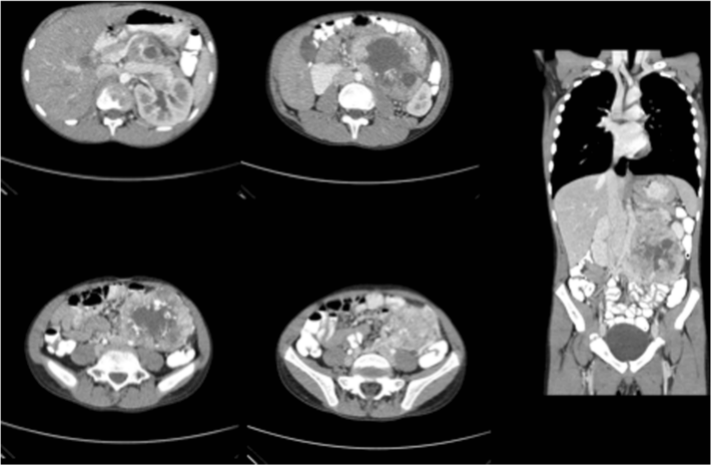
Computerized tomography of the abdomen revealing a congenital solitary left kidney and a 12-cm heterogeneous, markedly hypervascular mass located on the left paraaortic retroperitoneum with apparent involvement of the renal artery

A 12-year-old male presented with left flank pain for the past 3 months. He noticed an abdominal mass growth during the last month along with a left scrotal swelling. He denied having headaches, palpitations, or high blood pressure. Physical examination revealed a left upper quadrant abdominal mass and left varicocele. The patient had no clinical signs of catecholamine release, and this tumor was considered biologically inactive despite a slightly elevated serum dopamine (414 U/ml—normal range 65–400). Therefore, no preoperative alpha-adrenergic blockade was done, and the patient remained hemodynamically stable throughout the surgery. A computerized tomography (CT) scan of the abdomen showed a congenital solitary left kidney and a 12-cm heterogeneous, markedly hypervascular mass located on the left paraaortic region with apparent involvement of the renal artery (Fig. 5). A percutaneous transabdominal biopsy was performed and diagnosed PGL.

**Fig. 6:**
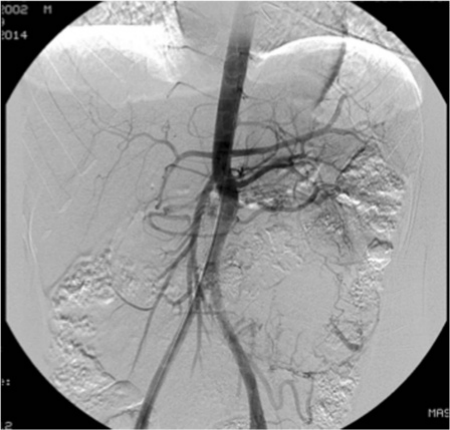
Arteriography showing arterial vascularization of the mass before the embolization

Preoperative scanning with 123-metaiodobenzylguanidine (I-123-MIBG) revealed no abnormal uptake. Arteriography was performed to identify blood supply to the tumor (Fig. 6). Aiming to minimize blood loss and facilitate resection, preoperative embolization of the two major feeding vessels arising from the inferior mesenteric artery and a left subcostal artery was performed similarly as described in case 1. However, a decrease of less than 50 % of the tumor vascularization was observed. In anticipation of a potential catecholamine crisis, an anesthesiology team was present with the patient intubated, under full venous access. The procedure was carried out 24 h before surgery, and the patient was maintained under continuous monitoring. The patient remained hemodynamically stable throughout the embolization and overnight, but referred progressive abdominal pain.

At surgery, preoperative ureteral stenting was performed in order to help identification during tumor dissection and prevent ureteral injury. Nevertheless, the possibility of autotransplantation of the solitary kidney was considered. A subcostal Chevron incision was made with extension to the left. The first surgical finding was extensive left colon necrosis, which was dissected from the anterior face of the mass, and resected completely at the end of the procedure, a right-sided Hartmann colostomy being performed.

**Fig. 7:**
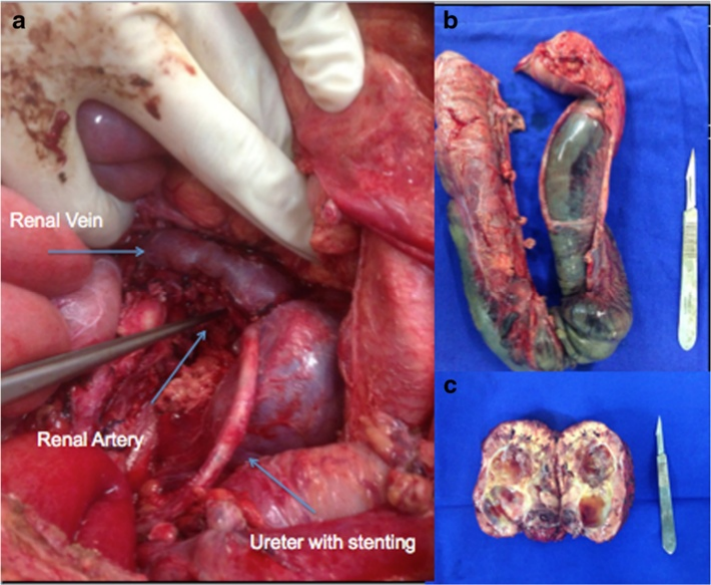
Images of surgical specimens and surgical site after mass removal. (**a**) surgical site with arrows indicating left renal vein with, left renal artery and left ureter; (**b**) surgical specimen of retosigmoidectomy demonstrating extensive left colon necrosis; (**c**) retroperitoneal mass after resection

The mass was exposed by mobilizing the splenic flexure and left colon medially. It was located medial to the left kidney and ureter and pushed the renal artery, as well as the aorta medially, being partially adherent to all those structures. Careful and tedious dissection liberated the tumor completely from the neighboring structures, requiring intermittent aortic and iliac clamping, with estimated blood loss of 1200 ml that was treated with three blood cell packs. Despite the bleeding, the patient remained stable during the whole procedure, without any hypertensive crisis. Pathological evaluation revealed that the specimen measured 13.0 × 10.0 × 4.0 cm and weighed 471 g. Sectioning revealed a multilobular, heterogeneous tumor mass with foci of hemorrhage without necrosis. Microscopically, the tumor consisted of nests of ovoid cells with nuclear enlargement. Chromogranin A and synaptophysin stains were both positive in the tumor cells, confirming the diagnosis of extra-adrenal retroperitoneal paraganglioma. Ki-67 stain was also positive. Mitotic index was extremely low and there were no signs of extracapsular invasion. Images of surgical specimens and surgical site after mass removal are demonstrated in Fig. 7.

**Fig. 8:**
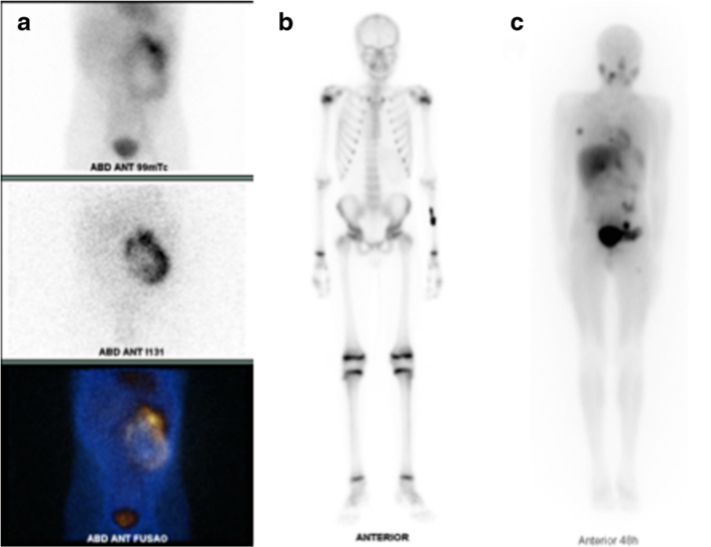
Preoperative MIBG scanning showing retroperitoneal mass with abdominal uptake (**a**). Bone scan with new uptake on the left forearm (**b**). Postoperative MIBG scanning with the right costal arch and left iliac bone uptake (**c**)

The patient had a good postoperative recovery with functioning colostomy and no renal function impairment and was discharged on the seventh postoperative day after removal of the ureteral stent. After 6 weeks, the patient had a normally functioning colostomy, but became acutely oliguric. A mid-ureteral stenosis was identified, requiring an emergency ureteral stenting. Follow-up evaluation with MIBG scan and bone scintigraphy also depicted multiple areas of intense activity suggestive of metastatic disease in the left forearm, right costal arches, and left iliac bone who were absent in preoperative staging. Figure 8 shows pre- and postoperative MIBG scanning. The patient was referred for genetic testing and is currently being treated with systemic chemotherapy.

### Discussion

The diagnosis of PGL can be difficult in cases presenting as a non-secreting abdominal mass. It can be found during the monitoring of a patient with a predisposition syndrome or incidentally before clinical manifestations related to hypersecretion of catecholamines [6]. Hypertension secondary to secretion of adrenaline and noradrenaline is found more frequently in pediatric patients, accounting for 60 to 90 % of cases as opposed to 50 % in adults [7, 8]. The PGL might be responsible for 1 % of all cases of pediatric hypertension. If PGL exclusively secretes dopamine, which occurs in many malignant cases, blood pressure is normal and the diagnosis is difficult [9].

Well-established hereditary disorders associated with PGL are MEN 2A and 2B, von Hippel-Lindau syndrome and neurofibromatosis type 1 [5, 10, 11]. Germ-line mutation is found in approximately 56 % of patients with PGL before 18 and 70 % of children when the disease becomes apparent before 10 years of age [12]. The discovery of a multifocal involvement of PGL is highly suggestive of a familial genetic disease. Twelve susceptibility genes have been identified in PGL:VHL gene, RET (involved in MEN2), NF1 (associated with neurofibromatosis type 1) SDHAF2, SDH-A, B, C, or D (four subunits of mitochondrial succinate dehydrogenase) TMEM127, MAX, HIF2A, and FH [4, 5]. Given the high percentage of mutations identified in patients with apparently sporadic presentation of the disease, and particularly in the pediatric population, some authors recommend that any patient with PGL should be submitted to genetic testing [13]. Of the 12 known susceptibility genes, six (RET, VHL, NF1, SDHB, SDHC, and SDHD) must be immediately investigated in any patient with PGL. Monitoring of blood pressure and annual measurement of serum and urinary metanephrines are recommended (MEN2, VHL, and SDH mutation). Patients with VHL should also benefit from a fundus and abdominal ultrasound or a thoracoabdominal and pelvic CT/MRI regularly [4].

In the pediatric population, elevated plasma-free and fractionated metanephrines have a high sensitivity close to 100 % and a specificity of 95 %. A concentration of plasma-free metanephrine greater than four times the standard was uniformly associated with the presence of a tumor [14]. Vanillylmandelic acid assay (VMA) is not recommended because of poor sensitivity [13]. Nevertheless, there is currently no formal recommendation on the type of examination to be performed. Combining a plasmatic test and a urinary test allows good sensitivity and specificity [5].

Abdominal CT scan, magnetic resonance imaging (MRI), metaiodobenzylguanidine (MIBG) scintiscan, and octreotide scan (Octreoscan) have all been described as means of identifying PGL [15]. As long as there is no contraindication, a contrast-enhanced abdominal CT should be the initial imaging modality because it is readily available and highly sensitive. In patients with renal failure or contrast allergy, an MRI can be obtained. Nuclear medicine studies are reserved for patients with biochemical evidence of disease but with a negative CT or MRI and in suspected cases of metastatic disease. Even though there is no pathognomonic feature on CT scan to identify PGL, a heterogeneous, hypervascular mass with differential enhancement indicating areas of necrosis arising from the retroperitoneum is highly predictive [3].

Some features such as central necrosis, high mitotic index, large size (greater than 5 cm), capsular rupture, or vascular invasion suggest malignancy. The only formal criterion for malignancy is the presence of metastasis in a site that does not contain chromaffin tissues (lymph node, liver, bone, lung). An important feature in pediatrics is the possibility of developing such metastases years after the discovery of the primary tumor, which warrants long-term surveillance, in particular, through an annual determination of urinary catecholamines [15]. In the pediatric population, about 12 % of PGL are malignant. This proportion can reach 46 % in some series [8].

Possible biochemical indicators of malignant disease include dopamine hypersecretion or markedly elevated plasma or urinary metanephrines. The most well-defined genetic risk factor for malignant disease is a mutation on the SDHB gene, which is clinically associated with an earlier onset of disease and more aggressive malignancy. Often, metastatic disease is related to retroperitoneal location (69 %) and is associated with SDHB mutation (72 versus 10 %). SDHD mutations are more frequently found in the cephalic or cervical tumors. These results should be considered in clinical practice especially during the etiologic diagnosis and monitoring. Given the possibility of very late metastases, genetic investigation is of major importance because it allows to identify patients with a high risk of developing recurrence and metastasis and thus requiring prolonged surveillance. Children with a cervical spine tumor should primarily benefit from analysis of the SDHD gene and those with metastatic retroperitoneal form from a study of SDHB [5].

The management of malignant PGL is primarily surgical as resection is the only potentially curative treatment, but depends on tumor staging (localized versus metastatic). The overall prognosis for tumors with complete surgical resection is excellent [4]. Preoperative biopsy should not be routinely performed due to the risk of an adrenergic crisis [5]. Alpha-adrenergic blockade for at least 4 weeks preoperatively may be administered in order to prevent and, if needed, to treat intra-operative catecholamine release [1, 3]. As neither signs nor symptoms of catecholamine release were observed in both cases prior to surgery and during biopsy, no alpha-adrenergic blockade was performed and no systemic repercussions were observed.

Initial complete resection with intent to cure has been shown to improve survival, while surgical debulking is often used in an attempt to achieve biochemical control, improve response to systemic therapies, palliate symptoms, or simply to decrease tumor burden. Debulking procedures in patients with extra-abdominal metastases should be employed only for palliation and not for biochemical remission. Conversely, a more aggressive operative approach should be offered to patients with exclusively intra-abdominal disease with a goal of postoperative biochemical remission when a complete resection (RO/R1) is possible [2].

The surgical technique depends on the tumor location. Laparoscopy can also be offered in cases of abdominal localization [3]. However, there are no data on the benefits of aggressive resection or debulking in the setting of locally invasive, metastatic, or recurrent disease [2].

Preoperative embolization is not routinely utilized because of the theoretical concern that blocking the arterial supply will induce ischemia and cell death, releasing catecholamines into an open venous outflow leading to hypertensive crisis. However, preoperative embolization has been described in the surgical management of carotid body tumors where it was shown to decrease intra-operative blood loss and time of surgery without complications [16]. It can be performed under intensive monitoring to reduce tumor vascularity of large tumors and hence facilitate surgical resection. Although embolization is effective in decreasing blood loss and operative time in patients with massive pheochromocytoma, in paraganglioma cases, it is a matter of debate [1]. Based in our small experience of two cases, one with one major complication (intestinal ischemia) and another with extensive blood loss despite embolization, we consider it of limited effectiveness.

Surgical ablation of metastasis should be considered whenever possible. The use of percutaneous techniques (radiofrequency, cryotherapy, alcohol injection) is a possible means to obtain local control in more than 50 % of cases, or at least temporarily reduce symptoms. The choice of the appropriate technique depends on the child’s age, clinical presentation, location and number of metastasis [17, 18].

Currently, the use of MIBG therapy with I^131^ seems to be the most effective treatment in adults. This is a complex treatment that requires a number of prerequisites such as MIBG injury setting and hematopoietic stem cell availability. The procedure is particularly of limited value in pediatrics because of the high radiation dosage [19]. External radiation is not very effective on PGL and requires high doses (40–50 Gy). Its use should be reserved for the treatment of painful bone metastases to relieve symptoms. External radiation is known to destroy the tumor uptake of MIBG and as a result, these irradiated tumors no longer benefit from the metabolic irradiation with I-131-MIBG. Chemotherapy is recommended for patients in whom tumor size reduction could make it amenable to surgery or those with general symptoms that chemotherapy might improve. Chemotherapy can cause hypertensive crisis, and preventive treatment must always precede the first course [20, 21].

In the presence of metastatic PGL, overall life expectancy at 5 and 10 years is estimated at 78 and 31 %, respectively, but is very poorly evaluated in the literature [15]. The prognosis also depends on the metastatic localization, with a life expectancy of less than 5 years for hepatic or pulmonary disease. Studies indicate that patients without extra-abdominal disease are more likely to achieve both remission and a durable biochemical response from aggressive resection of metastatic paraganglioma, and debulking operations are unlikely to lead to sustained control of catecholamine secretion or pharmacologic independence [2, 22].

## Conclusions

Many studies in the adult population have established recommendations for the diagnosis and therapeutic management of PGL, but few studies concern the pediatric population. Because malignant PGL is more important in the pediatric population, screening and early diagnosis of PGL is advisable in children with genetic predisposition. Better understanding of genetics allows a better assessment of risk metastatic and therefore prognosis, especially in familial forms. Surgical resection is the mainstay of treatment and should be performed despite the difficulties involved in the procedure. The role of preoperative embolization is not established, and in our experience, it has provided little benefit and major complications.

The complexity of this pediatric pathology requires a multidisciplinary management involving pediatricians, endocrinologists, pediatric surgeons, urologists, anesthesiologists, geneticists, radiologists, nuclear medicine physicians, and oncologists.

### Consent

Written informed consent was obtained from the patients for publication of this case report and any accompanying images.

## Abbreviations

CT: computerized tomography
MEN: multiple endocrine neoplasm
MIBG: metaiodobenzylguanidine
MRI: magnetic resonance imaging
PGL: paraganglioma
SDH: succinate dehydrogenase
VHL: Von Hippel-Lindau
